# Microbial carbon fixation pathways shifts during artificial *Haloxylon ammodendron* restoration with clay sand barriers in arid deserts: a metagenomic analysis

**DOI:** 10.3389/fmicb.2026.1884493

**Published:** 2026-07-27

**Authors:** Hao Wu, Da-cheng Song, Ze Yao, Qi Wang, Zi-zhu Yan, Fang-lan He, Shu-jiang Guo, Li-de Wang

**Affiliations:** 1 Gansu Desert Control Research Institute, Lanzhou, China; 2 Gansu Linze Desert Ecosystem Observation and Research Station, Linze, China; 3 Gansu Hexi Corridor Forest Ecosystem Research Station, Minqin, China

**Keywords:** carbon fixation, functional genes, metagenome, microbial diversity, soil carbon sequestration, vegetation restoration

## Abstract

Soil microbial carbon fixation is influenced by the combined effects of microbial community composition, functional gene distribution, and environmental factors, and is closely associated with vegetation restoration processes. However, the soil carbon fixation process and its coupling mechanisms mediated by microorganisms at different vegetation restoration stages in arid regions remain unclear. In this study, we applied metagenomic sequencing to investigate soil from a clay sand barrier *Haloxylon ammodendron* sand-fixing restoration area at the southeastern edge of the Badain Jaran Desert, spanning a 60-year vegetation restoration time sequence (1, 5, 10, 20, 40, and 60 years) and shifting sand as a control. We explored the impacts of vegetation restoration and its long-term sequence on soil properties, microbial community structure, carbon fixation genes, and carbon fixation pathways. The results showed that vegetation restoration improved regional soil nutrient levels and organic carbon accumulation, with these positive effects progressively amplified over the restoration time sequence. Additionally, vegetation restoration not only reshaped microbial community composition but also induced changes in carbon fixation-related genes and pathways. A 10-year restoration period served as a critical time point, with microbial community diversity and carbon fixation gene abundance exhibiting pronounced fluctuations during the first 10 years, followed by relative stabilization thereafter. This threshold likely reflects a transition from intense plant-microbe competition to a more balanced coexistence as vegetation succession progresses and soil conditions stabilize. Among the six major microbial carbon fixation pathways, the rTCA cycle had the highest relative gene abundance, making it the dominant carbon fixation pathway in the region. Soil properties, particularly soil water content (SWC) and total phosphorus (TP), were identified as critical factors influencing both microbial community composition and carbon fixation-related genes. These findings suggest that clay sand barrier *Haloxylon* restoration not only fulfills its role in sand stabilization but also alters the soil environment, driving a functional shift in the microbial community from autotrophic to heterotrophic processes. This study deepens our understanding of soil carbon fixation processes in arid desert ecosystems and provides theoretical guidance for carbon management in similar arid regions.

## 1 Introduction

Drylands are an essential component of the Earth System and are especially vulnerable to climate change and land degradation (Lian et al., 2021). Accounting for approximately one-quarter of the global terrestrial surface, deserts hold substantial yet underexplored potential for carbon sequestration (Obalum et al., 2019). Despite the inherently low organic carbon concentrations in arid soils, these regions possess significant carbon reservoirs, where even minor shifts in carbon inputs or outputs can exert profound effects on soil quality and the global atmospheric CO_2_ balance (Davidson and Janssens, 2006). However, our understanding of the metabolic functions of soil microbial communities and their responses to vegetation restoration in arid desert environments remains insufficient and fragmented (Li et al., 2024).

Plant-derived carbon serves as the foundational source of soil organic carbon, and increasing plant inputs is widely acknowledged as a critical pathway to enhance soil carbon sequestration (Feng et al., 2024). In arid regions, vegetation restoration profoundly influences soil carbon dynamics by augmenting plant carbon inputs and improving soil structure. This process not only serves as an effective method for rehabilitating degraded or eroded soils but also represents a pivotal strategy for bolstering soil carbon storage, maintaining global carbon equilibrium, and mitigating climate change (Han et al., 2023). Furthermore, vegetation restoration can directly increase soil organic carbon stocks while indirectly stabilizing carbon pools by regulating carbon turnover rates and microbial functional activities (Fanin and Bertrand, 2016). Nonetheless, it is crucial to recognize that increasing plant-derived carbon inputs does not universally enhance soil carbon sequestration. In nutrient-limited soils, additional carbon inputs may intensify microbial “mining effects” on soil organic nutrients, leading to accelerated decomposition of native organic matter and, paradoxically, a reduction in net carbon accumulation (Zhou et al., 2022). While significant progress has been made in recent years in elucidating the potential of vegetation restoration to enhance soil carbon sequestration, most research has narrowly focused on patterns of carbon stock increases, with relatively limited attention to the underlying mechanistic drivers (Yang Y. et al., 2024; Yang Z. et al., 2024).

Microbial communities are integral to global biogeochemical processes, mediating the cycling of carbon, nitrogen, and sulfur between the Earth and atmosphere, and they play a critical role in maintaining the stability and functionality of terrestrial ecosystems (Elbert et al., 2012). Despite the extreme environmental conditions in arid desert habitats, various autotrophic microorganisms have evolved highly specialized genetic traits and metabolic pathways to adapt to these stressors, demonstrating exceptional resilience and substantial carbon fixation potential (Chang et al., 2023). Functional genes associated with microbial carbon fixation have been widely detected in soils across diverse arid regions, underscoring the significance of these microorganisms in carbon cycling (Jordaan et al., 2020). In recent years, an arena that has been a major focus of such studies is the soil microbiome and its push-pull relationship with biogeochemical cycling (Abs et al., 2022). However, the mechanisms regulating microbial carbon fixation capacity remain highly debated (Dong et al., 2024). Some studies highlight the abundance and diversity of carbon-fixing microorganisms as the primary determinants of carbon fixation potential, while others emphasize the overriding influence of abiotic soil factors (Lynn et al., 2017; Xiao et al., 2018). Additionally, the accumulation of microbial necromass has emerged as a potentially critical driver of soil carbon cycling, with its impact potentially rivaling that of living microorganisms (Angst et al., 2021). To date, six principal carbon fixation pathways have been identified: the Calvin cycle is considered the most dominant carbon fixation pathway in nature, particularly in nutrient-rich environments. However, its high energy demand significantly constrains its activity in arid desert ecosystems (Claassens et al., 2016). In contrast, low-energy-demand pathways, such as the rTCA cycle and DC/4-HB cycle, are more frequently observed in arid deserts (Fuchs, 2011). The 3-HP bi-cycle and 3-HP/4-HB cycle both exhibit strong adaptability to arid and nutrient-poor environments; however, the 3-HP/4-HB cycle is better suited to high-temperature and high-salinity conditions, whereas the 3-HP bi-cycle is more common in soils with moderate salinity (Hügler and Sievert, 2011). Although the WL pathway requires minimal energy input, its strict dependence on anaerobic conditions limits its distribution in sandy desert soils with high sand content (Ragsdale and Pierce, 2008). Based on the low-energy environment of arid regions, we hypothesized that the rTCA cycle would dominate microbial carbon fixation, rather than the energetically costly Calvin cycle. Overall, microorganisms adapt to environmental stressors by optimizing their metabolic pathways. Understanding the dynamic shifts of microbial carbon fixation strategies in arid desert soils and their contributions to the carbon cycle is of critical ecological significance.

The remarkable spatiotemporal variability in soil physico-chemical properties has promoted microorganisms to evolve a wealth of different strategies to cope with the most extreme conditions. Evidence is growing that these microbially mediated modifications of soil properties can have ecological ramifications (Philippot et al., 2024). However, the primary factors driving microbial community functions remain debated across different ecosystems and soil conditions. For example, in desert regions, low soil water content limits solute availability and absorption by both plants and microbial communities, negatively impacting processes such as carbon fixation, denitrification, and the uptake and transport of phosphorus (Li et al., 2024). Soil available phosphorus (AP) has been shown to enhance soil carbon and sulfur cycling, making it one of the most critical factors driving microbial functions and improving soil fertility (Ding et al., 2021). Additionally, soil pH plays a pivotal role in regulating the relationship between soil inorganic carbon (SIC) and soil organic carbon (SOC) under arid conditions (Naorem et al., 2022). Therefore, further studies are needed to disentangle the specific drivers influencing microbial community structure and function under varying soil conditions.

The southeastern edge of the Badain Jaran Desert represents a typical ecologically fragile zone. Harsh arid conditions, sparse vegetation, and loose surface materials resulting from water scarcity have rendered this region a major source of sandstorms within China, as well as a primary pathway for transboundary dust storms (Ma et al., 2021). To mitigate wind erosion, extensive *Haloxylon* afforestation projects have been implemented across the region. However, ecological restoration is not merely a simple reverse process. Under natural conditions, the time required for artificially restored ecosystems to attain a complete functional structure often significantly exceeds the implementation timeframe of ecological restoration projects (Zhao et al., 2018). Previous studies have predominantly focused on evaluating the windbreak efficiency, species succession, and soil nutrient distribution of this model, with limited attention given to the carbon transformation processes regulated by soil microorganisms during the different stages of *Haloxylon* restoration (Sun et al., 2012; Ding et al., 2018; Song et al., 2024). Furthermore, the effects of soil stoichiometric changes on the carbon fixation potential of microbial communities remain poorly understood. This study employed metagenomic sequencing to investigate soil samples from *Haloxylon* afforestation areas along a 60-year restoration chronosequence in the southeastern edge of the Badain Jaran Desert. By examining changes in soil microbial community composition and annotating functional genes involved in carbon fixation, this study aimed to elucidate the critical roles of microbial taxa across different vegetation restoration stages (0, 1, 5, 10, 20, 40, and 60 years). The following scientific questions were addressed: (1) Does vegetation restoration lead to a sustained increase in soil organic carbon? (2) What are the predominant carbon fixation pathways utilized by soil microorganisms in arid desert regions? (3) Which soil physicochemical properties exert the most significant influence on microbial carbon fixation variability? The findings of this study will advance our understanding of microbial-mediated carbon fixation processes in arid desert soils and provide theoretical guidance for carbon management strategies in arid ecosystems.

## 2 Materials and methods

### 2.1 Study site and sample collection

The study area (102°55′-102°58′E, 38°34′-38°37′N) is located in the Minqin Desert region, on the southeastern margin of the Badain Jaran Desert in Northwest China (Table 1). Historically, this region was characterized by extensive water bodies prior to the Han Dynasty; however, centuries of environmental degradation, driven by unsustainable land use and climatic changes, have resulted in severe desertification. The region experiences a temperate continental arid desert climate, with an average annual temperature of 7.6 °C and a frost-free period of approximately 176 days. Annual precipitation averages 110 mm, with nearly 60% of the rainfall occurring between July and September. In stark contrast, the mean annual evaporation is approximately 2,600 mm, exceeding precipitation by a factor of 23. Groundwater levels are typically 16–20 m below the surface, and the region is frequently subjected to intense aeolian processes, including wind erosion and sandstorms. The average annual wind speed is 2.5 m/s, with an average of 25.1 days of strong winds, 25.6 days of sandstorms, and 27.8 days with wind speeds exceeding level eight on the Beaufort scale (Zhao et al., 2023). The predominant soil types in the region include gray-brown desert soil, aeolian sandy soil, and meadow soils. Vegetation is sparse and dominated by drought-tolerant shrubs, semi-shrubs, and herbs, forming a simple and open community structure. The dominant plant species include *Haloxylon ammodendron*, *Reaumuria songarica*, *Nitraria tangutorum*, *Calligonum mongolicum*, *Artemisia arenaria*, *Agriophyllum squarrosum*, and *Salsola collina* (Zhao et al., 2022; Man et al., 2023).

**Table 1:** Detailed information on sample plots across different vegetation restoration stages in the study area.

Restoration years	Longitude	Latitude	Elevation (m)	Main vegetation species	Shannon diversity	Simpson diversity	Soil types
0	102°57′24″E	38°36′39″N	1,324	*Nitraria tangutorum*	0.14 ± 0.22e	0.08 ± 0.12c	Aeolian sandy soil
1	102°57′28″E	38°35′59″N	1,321	*Nitraria tangutorum* *Haloxylon ammodendron*	0.42 ± 0.28 cd	0.36 ± 0.26b	Aeolian sandy soil
5	102°55′40″E	38°37′34″N	1,318	*Nitraria tangutorum* *Haloxylon ammodendron*	0.78 ± 0.39b	0.50 ± 0.22ab	Aeolian sandy soil
10	102°57′22″E	38°36′35″N	1,320	*Salsola collina* *Haloxylon ammodendron*	0.53 ± 0.49bcd	0.33 ± 0.28b	Aeolian sandy soil
20	102°57′45″E	38°36′10″N	1,322	*Calligonum mongolicum* *Haloxylon ammodendron*	1.10 ± 0.32a	0.64 ± 0.10a	Aeolian sandy soil
40	102°57′53″E	38°36′01″N	1,317	*Calligonum mongolicum* *Haloxylon ammodendron*	0.67 ± 0.39bc	0.41 ± 0.28b	Aeolian sandy soil
60	102°58′30″E	38°34′49″N	1,330	*Haloxylon ammodendron*	0.37 ± 0.37de	0.37 ± 0.37b	Aeolian sandy soil

In late August 2024, seven experimental treatments were established to investigate the responses of soil microbial carbon fixation pathways and their influencing factors during vegetation restoration. These treatments included six *Haloxylon* restoration sites with clay-sand barriers that had been deployed for varying durations (1, 5, 10, 20, 40, and 60 years) and one control site located in a nearby mobile sandy area without restoration. All experimental plots were situated in the northwest section of the Minqin Desert Control Comprehensive Experimental Station. The plots were flat, with consistent elevations and landforms, and adjacent plots were separated by a minimum distance of 1 km to minimize potential cross-contamination. The clay-sand barriers were constructed using locally sourced adhesive clay from Minqin County and were arranged in a belt-like configuration perpendicular to the prevailing wind direction. Each barrier was approximately 30 cm in height, with a row spacing of 3 m. One-year-old *Haloxylon* seedlings were artificially planted between the barriers during their establishment and irrigated to ensure initial survival. After establishment, no further management or maintenance was conducted. The duration of the clay-sand barrier deployment corresponded to the planting years of *Haloxylon*.

In early September 2024, five 1 m × 1 m plots were randomly established at each experimental site, maintaining a minimum distance of 10 m between plots to account for spatial heterogeneity. Within each plot, three replicate soil samples were collected from the 0–10 cm soil layer, resulting in a total of 15 soil samples per site. These samples were composited and thoroughly homogenized to produce a single pooled soil sample for each site. In total, 105 soil samples were collected across the chronosequence. The samples were sieved on-site through a 2 mm mesh and immediately transported to the laboratory in an ice box to preserve microbial activity. Each composite soil sample was subsequently divided into three subsamples: one was placed in sterile tubes and stored at −80 °C in an ultralow temperature freezer for metagenomic sequencing analysis, the second was stored at 4 °C for the determination of soil dissolved organic carbon (DOC) and microbial biomass carbon (MBC), and the third was air-dried for the analysis of soil physicochemical properties.

### 2.2 Determination of soil properties

A comprehensive array of analyses was conducted to elucidate the physicochemical and biological characteristics of the soil. Total carbon (TC) and soil organic carbon (SOC) concentrations were quantified using an Elementar vario MACRO cube C/N analyzer (Elementar Trading Co. Ltd., Germany). Total nitrogen (TN) and total phosphorus (TP) content were assessed with a CleverChem Anna autoanalyzer (DeChem-Tech GmbH, Germany). Microbial biomass carbon (MBC) was estimated via the chloroform fumigation-extraction method. Dissolved organic carbon (DOC) was quantified according to the methodology established by Jones and Willett (2006), which provides precise estimates of labile carbon pools critical for understanding microbial and biochemical processes in soil. Briefly, 2.5 g of soil was shaken with 25 mL of distilled water (1:10 w/v soil-to-solution ratio) in 50 mL polypropylene bottles on a reciprocating shaker at 200 rpm for 15 min. The soil extracts were then centrifuged at 8000 × g for 10 min, and the supernatant was filtered through 0.45 μm membrane filters. The filtrate was analyzed for dissolved organic carbon using a GelDoc Dissolved organic carbon Analyzer (Bio-Rad Trading Co. Ltd., China). Bulk density (BD) was determined using the cutting ring method with 100 cm^3^stainless steel rings. Particle size distribution, encompassing clay, silt, and sand fractions, was analyzed through the wet dispersion method utilizing a Mastersizer 2000 laser grain size analyzer (Malvern Instruments Ltd., UK). Soil water content (SWC) was measured gravimetrically by drying soil samples to a constant weight at 105 °C. Soil pH was determined with a PHS-3C pH meter (Shanghai INESA Scientific Instruments Co. Ltd., China). Electrical conductivity (EC) was assessed using a 307A conductivity meter (Nanjing Teai-de Instrument & Equipment Co. Ltd., China).

### 2.3 DNA extraction, library construction, and metagenomic sequencing

Genomic DNA was extracted from soil samples using the CTAB method under a standardized protocol to ensure high-purity and high-integrity DNA suitable for downstream applications. Briefly, 0.5 g of soil was lysed in 1000 μL of CTAB lysis buffer (containing lysozyme) and incubated at 65 °C for 1 h with intermittent vortexing to facilitate complete cell lysis. The lysate was centrifuged at 12000 rpm for 10 min, and the supernatant was subjected to a two-step purification process. First, phenol:chloroform: isoamyl alcohol (25:24:1) was used to remove proteins and other impurities, followed by chloroform: isoamyl alcohol (24:1) to eliminate residual organic contaminants. DNA was precipitated by adding isopropanol and incubating at −20 °C for 30 min, then pelleted by centrifugation at 12000 rpm for 15 min. The pellet was washed twice with 75% ethanol, air-dried at room temperature, and resuspended in sterile nuclease-free water (ddH₂O). DNA quality and integrity were assessed using the Agilent 5,400 system, which evaluates DNA fragment size and purity. DNA concentration was quantified using a Qubit 4.0 fluorometer (Thermo Fisher Scientific Inc., USA).

Library preparation was performed using the NEBNext® Ultra™ DNA Library Prep Kit for Illumina (New England Biolabs Inc., USA), following the manufacturer’s protocol to ensure reproducibility and standardization. Genomic DNA was fragmented to an average size of approximately 350 bp using a Covaris S220 ultrasonicator (Covaris Inc., USA). Fragmented DNA underwent end-repair, A-tailing, and ligation with Illumina-compatible adapters. PCR amplification was subsequently performed to enrich adapter-ligated DNA under optimized conditions, followed by a second purification step to eliminate non-specific amplification products and adapter dimers. The quality of constructed libraries, including fragment size distribution and overall integrity, was assessed using the Agilent 5,400 system. Library concentrations were quantified using qPCR with the KAPA Library Quantification Kit (Roche Inc., Switzerland). Only libraries with a minimum concentration of 1.5 nM and an optimal fragment size distribution (350 bp) were selected for sequencing.

The libraries were pooled and sequenced on the Illumina NovaSeq platform using the PE150 paired-end sequencing strategy. Metagenomic sequencing was performed by Microeco Tech Co., Ltd. (Shenzhen, China). Raw sequencing data were stored in FASTQ format. The numbers of raw reads and clean reads for each sample are detailed in Table 2. The original contributions presented in the study are publicly available. The raw metagenomic sequencing data have been deposited in the Genome Sequence Archive (GSA) of the China National Center for Bioinformation (CNCB) under accession number CRA046182 (https://ngdc.cncb.ac.cn/gsa).

**Table 2:** Statistics of soil genomic DNA sequencing data.

Sample	InsertSize (bp)	SeqStrategy	Raw base (GB)	RawReads (#)	Raw Q20 (%)	Raw Q30 (%)	Clean reads (#)	Clean Q20 (%)	Clean Q30 (%)	Cleaned (%)
0 a-1	350	150:150	10.15	33,833,565	98.49	95.49	32,187,310	99.22	97.1	95.13
0 a-2	350	150:150	11.28	37,599,452	98.52	95.61	35,892,147	99.24	97.18	95.46
0 a-3	350	150:150	12.09	40,298,946	98.49	95.53	38,322,548	99.24	97.19	95.1
1 a-1	350	150:150	10.26	34,204,878	98.47	95.41	32,420,682	99.21	97.05	94.78
1 a-2	350	150:150	12.16	40,535,129	98.51	95.54	38,585,048	99.23	97.13	95.19
1 a-3	350	150:150	13.94	46,451,666	98.5	95.55	44,213,818	99.25	97.22	95.18
5 a-1	350	150:150	11.47	38,224,519	98.27	94.82	36,420,078	99.09	96.64	95.28
5 a-2	350	150:150	14.58	48,607,651	98.23	94.94	46,268,943	99.07	96.77	95.19
5 a-3	350	150:150	13.98	46,598,412	98.43	95.35	43,868,258	99.23	97.14	94.14
10 a-1	350	150:150	12.82	42,719,840	98.2	94.66	40,179,815	99.06	96.6	94.05
10 a-2	350	150:150	13.87	46,229,423	98.76	96.17	44,703,348	99.33	97.41	96.7
10 a-3	350	150:150	11.33	37,755,144	98.18	94.68	36,025,878	99.1	96.65	95.42
20 a-1	350	150:150	10.38	34,588,800	98.25	94.84	32,923,315	99.13	96.75	95.18
20 a-2	350	150:150	12.32	41,068,540	98.27	94.79	39,414,292	99.01	96.4	95.97
20 a-3	350	150:150	11.53	38,439,359	98.54	95.42	34,034,105	99.33	97.3	88.54
40 a-1	350	150:150	13.63	45,421,097	98.59	95.84	43,379,774	99.31	97.41	95.51
40 a-2	350	150:150	12.13	40,445,418	98.59	95.79	38,614,686	99.28	97.32	95.47
40 a-3	350	150:150	11.56	38,528,522	98.27	94.8	36,426,949	99.07	96.63	94.55
60 a-1	350	150:150	12.98	43,255,036	98.55	95.69	41,261,163	99.27	97.28	95.39
60 a-2	350	150:150	13.94	46,480,301	98.58	95.79	44,500,293	99.29	97.33	95.74
60 a-3	350	150:150	11.20	37,329,019	98.56	95.72	35,590,585	99.28	97.32	95.34

### 2.4 Bioinformatics analysis

Raw sequencing data were quality-filtered using Trimmomatic (v0.39) with the following parameters: ILLUMINACLIP:adapters_path:2:30:10 (removal of adapter sequences), SLIDINGWINDOW:4:20 (trimming once the average quality score drops below 20 within a 4-bp sliding window), and MINLEN:50 (discarding reads shorter than 50 bp). After quality control, host-derived reads were identified and removed using Bowtie2 (v2.3.5.1) in very-sensitive mode, and the retained clean reads were used for subsequent analyses.

Taxonomic classification was performed using Kraken2 (v2.0.7) with a confidence threshold of 0.2, by aligning clean reads against a custom-built microbial nucleic acid database integrating the NCBI NT database and RefSeq sequences of bacteria, fungi, archaea, and viruses. The relative abundance of each taxon was then re-estimated using Bracken (v2.0) with default parameters (Brum et al., 2015). Based on the absolute abundance output from Bracken, the proportion of sequences assigned to each of the seven taxonomic levels (kingdom, phylum, class, order, family, genus, and species) relative to the total sequences per sample was calculated.

### 2.5 Statistical analyses

Levene’s test was applied to verify the homogeneity of variances. To assess significant differences in soil properties, microbial community diversity, carbon fixation pathways, and the abundance of carbon fixation functional genes across vegetation restoration stages, one-way analysis of variance (ANOVA) was performed, followed by Duncan’s multiple comparison test for *post-hoc* pairwise comparisons. Differences in microbial community composition were analyzed using Principal Coordinate Analysis (PCoA) based on Bray-Curtis dissimilarities, providing a clear visualization of community clustering patterns. The relationships between soil properties and carbon fixation functional gene abundance were explored using Pearson correlation analysis. To identify significantly differentially abundant carbon fixation genes across restoration stages, the Kruskal-Wallis rank-sum test was employed, and their contributions were further evaluated by Linear Discriminant Analysis Effect Size (LEfSe), using a strict LDA score threshold of 3.5 to quantitatively assess their relative importance. Redundancy analysis (RDA) was performed to quantify the influence of soil properties on the abundance of functional genes associated with key carbon fixation pathways, highlighting the environmental drivers of microbial functional potential. Data processing and organization were conducted in Microsoft Excel 2019, statistical computations were performed using SPSS 26.0, and visualization of results was implemented using Origin 2021, Canoco 5.0, and R packages such as “pheatmap,” “vegan,” and “ggplot2.”

## 3 Results

### 3.1 Changes in soil carbon content and soil properties at different restoration stages

**Figure 1:**
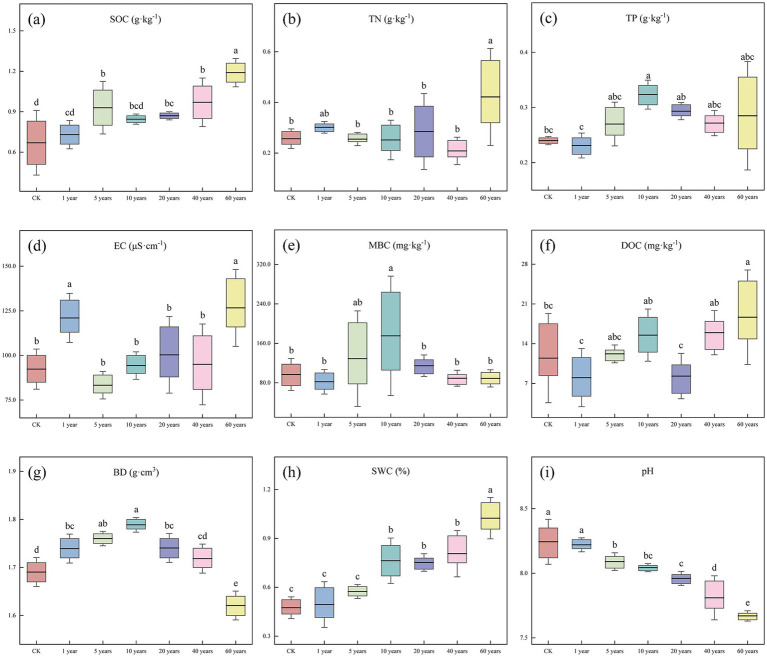
Changes in soil properties among the plots with different restoration stages. **(a)** Changes in SOC content across different planting years; **(b)** changes in TN content across different planting years; **(c)** changes in TP content across different planting years; (d) changes in EC across different planting years; **(e)** changes in MBC content across different planting years; **(f)** changes in DOC content across different planting years; **(g)** changes in BD across different planting years; **(h)** changes in SWC across different planting years; (i) changes in pH across different planting years. Significant differences between restoration stages are denoted by different lowercase letters (*p <* 0.05).

The variations in soil organic carbon (SOC) content and soil properties across sites with different vegetation restoration stages are presented in Figure 1. Compared with the control (CK), vegetation restoration significantly enhanced SOC content, which exhibited a fluctuating upward trend with increasing restoration years. Among all the sites, SOC content was lowest in CK (0.67 g/kg) and highest in the 60-year restoration site (1.19 g/kg), reflecting an increase of approximately 77.61% over the 60-year restoration period. Although the dissolved organic carbon (DOC) content declined during the initial stages of restoration, this trend gradually reversed as restoration progressed. Specifically, DOC content returned to the CK level after 5 years of restoration, continued to increase, and ultimately reached its maximum value of 18.66 mg/kg at the 60-year restoration site, approximately 1.63 times that of CK. In contrast to SOC and DOC, microbial biomass carbon (MBC) exhibited a unimodal pattern, characterized by an initial increase during the 1–10 year period, followed by a decline from 10 to 60 years. The MBC content peaked at 175.16 mg/kg in the 10-year restoration site, which was significantly higher than CK (*p <* 0.05), and subsequently decreased to match the CK level after 40 years of restoration.

The soil water content (SWC) in the 0–10 cm layer exhibited a consistent increasing trend, ranging from 0.47 to 1.02%. Among all sites, the SWC was lowest in CK and reached the highest value in the 60-year restoration site. Soil pH was highest in CK (approximately 8.24) and decreased progressively with vegetation restoration, ultimately declining to 7.67 in the 60-year restoration site. In contrast, total phosphorus (TP) content remained relatively stable across all sites, ranging from 0.23 to 0.32 g/kg. Total nitrogen (TN) and total carbon (TC), however, exhibited relative stability during the first 40 years of restoration, followed by a significant increase. By the 60-year restoration site, TN and TC reached 0.40 g/kg and 3.94 g/kg, respectively, values significantly higher than those in CK. Vegetation restoration also enhanced soil electrical conductivity (EC), with the most pronounced effects observed during the early (1 year) and late (60 years) stages of restoration. Bulk density (BD) increased slightly from 1.74 g/cm^3^ to 1.79 g/cm^3^ during the 1–10 year stage but subsequently declined, reaching its lowest value of 1.62 g/cm^3^ at the 60-year restoration site, which was significantly lower than CK. Additionally, vegetation restoration significantly increased the clay and silt content in the surface soil. Over the 60-year restoration period, compared with CK, the relative clay and silt contents increased by 866.67 and 1150.41%, respectively.

**Figure 2:**
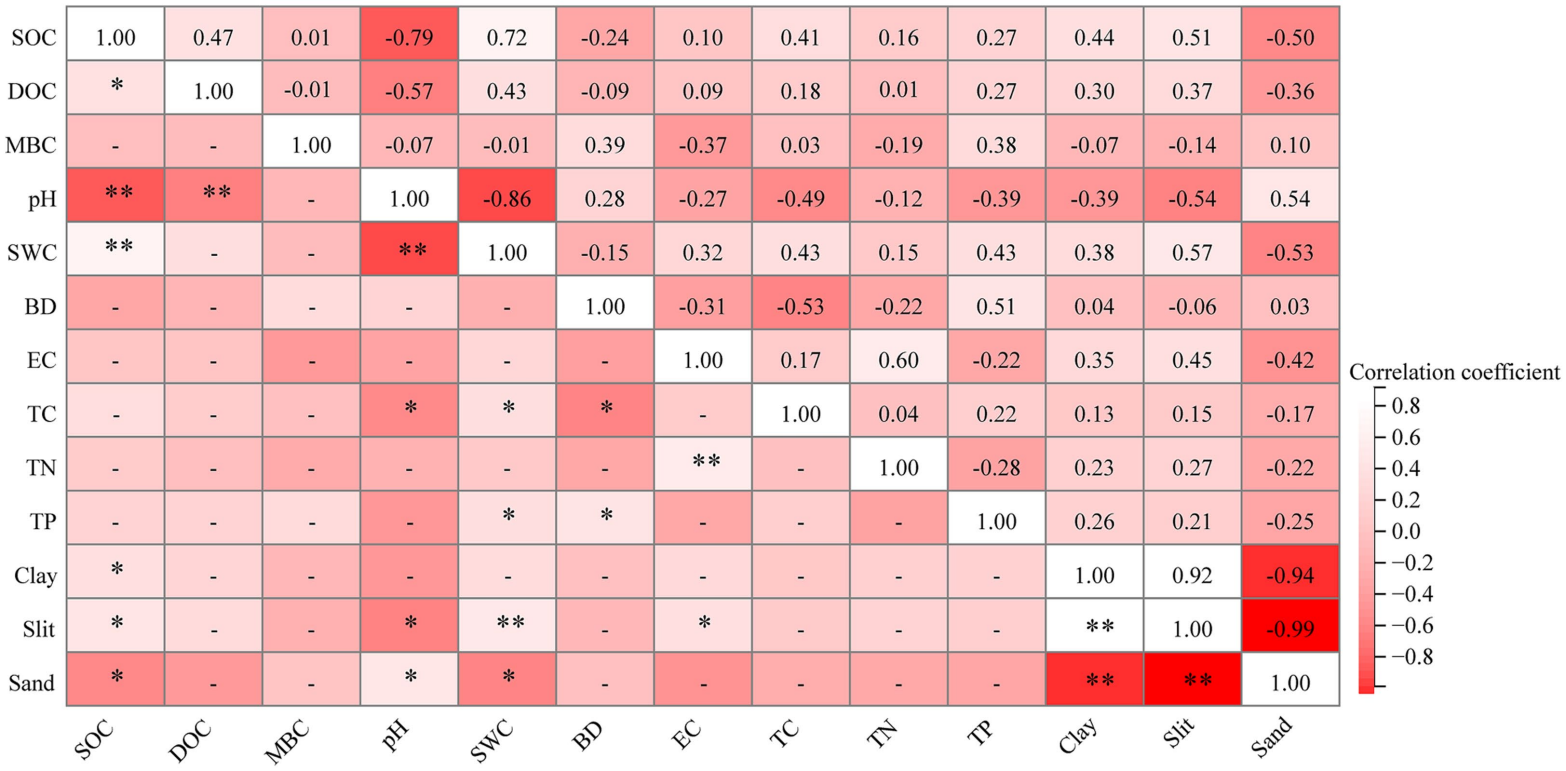
Pearson’ s correlation coefficient between soil properties. Analysis of the correlations between soil physicochemical properties, with significance levels denoted as *p < 0.05 and **p < 0.01.

Pearson’ correlation analysis revealed that SOC exhibited stronger correlations with regional soil physicochemical properties (Figure 2). Specifically, SOC was significantly positively correlated with clay, silt, and sand content (*p <* 0.05), highly significantly positively correlated with SWC (*p <* 0.01), and highly significantly negatively correlated with pH (*p <* 0.01). Similarly, DOC was highly significantly negatively correlated with pH (*p <* 0.01). In contrast, MBC showed no significant correlations with any soil physicochemical properties (*p* > 0.05).

### 3.2 Changes in microbial community composition at different restoration stages

The taxonomic results of metagenomic analysis at the kingdom-level are presented in Table 3. Across soil samples from the seven vegetation restoration stages, bacteria exhibited the highest relative abundance, making them the dominant microbial group in the region, accounting for 98.96–99.27% of the total microbial community. No significant differences in bacterial relative abundance were observed among samples (*p* > 0.05). Archaea ranked second, with a relative abundance ranging from 0.37 to 0.57%. Except for the control (CK), the relative abundance of archaea generally displayed a fluctuating upward trend as vegetation restoration progressed. Fungi exhibited a relatively low relative abundance, peaking at 0.53% in the 20-year restoration sample and reaching the lowest level (0.33%) in the 1-year restoration sample. No significant differences in fungal relative abundance were observed among the other samples (*p* > 0.05).

**Table 3:** Changes of relative abundance of kingdom-level microbe among the plots with different restoration stages.

Relative abundance (%)	CK	1 year	5 years	10 years	20 years	40 years	60 years
Bacteria	98.96 ± 0.07a	99.27 ± 0.03a	99.24 ± 0.21a	99.15 ± 0.04a	99.09 ± 0.04a	99.10 ± 0.03a	99.05 ± 0.06a
Archaea	0.54 ± 0.07ab	0.38 ± 0.03b	0.38 ± 0.10b	0.46 ± 0.04ab	0.37 ± 0.03b	0.54 ± 0.02ab	0.57 ± 0.06a
Fungi	0.49 ± 0.01ab	0.33 ± 0.01c	0.39 ± 0.11abc	0.38 ± 0.01abc	0.53 ± 0.04a	0.36 ± 0.01bc	0.37 ± 0.01bc

All values are presented as means ± SD (*n* = 3). Significant differences between restoration stages are denoted by different lowercase letters (*P <* 0.05).

**Figure 3:**
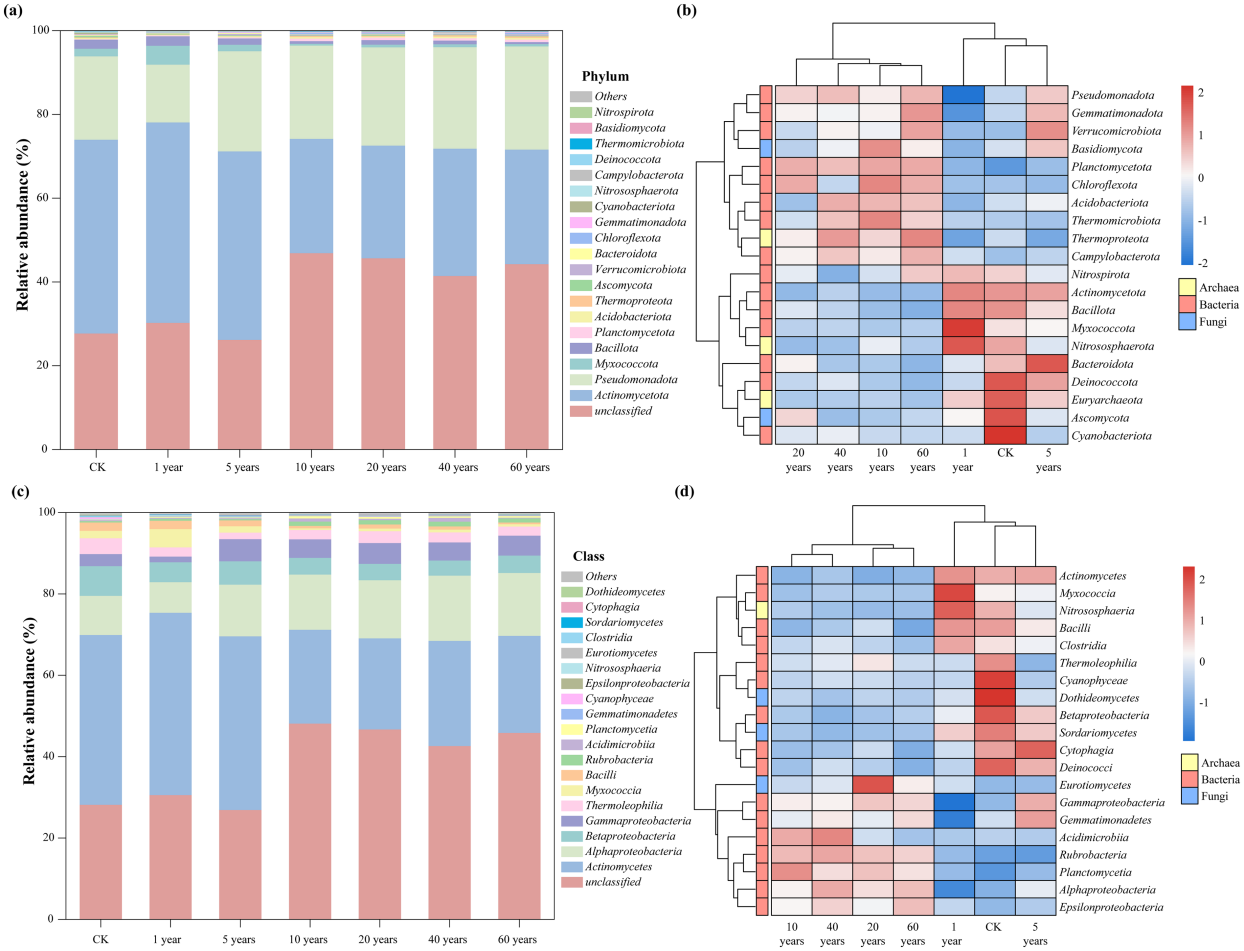
Changes in soil microbial community composition across different stages of vegetation restoration in the study area. Panels **(a,c)** illustrate the variations in the top 20 species across different taxonomic levels, including phylum, class. Panels **(b,d)** display heatmaps of Spearman correlations, highlighting the relationships in relative abundance rankings of the top 20 species at the corresponding taxonomic levels. Panel a shows a stacked bar chart of microbial phylum relative abundance over different restoration years, while panel b presents a clustered heatmap of phylum-level microbial community composition, colorcoded by domain. Panel c displays a stacked bar chart of microbial class relative abundance across the different restoration timeline, and panel d shows a corresponding heatmap of class-level composition, also clustered and colorcoded by domain. Each bar or heatmap column represents a restoration time point, with color legends indicating taxa and domains.

In this study, the top 20 dominant microbial taxa, based on relative abundance, were selected for further analysis at the phylum, class, genus, and species levels (Figure 3). At the phylum level, Actinomycetota and Pseudomonadota were the dominant bacterial phyla in the study area, with average relative abundances of 35.86 and 21.74%, respectively. The relative abundance of Actinomycetota gradually decreased with increasing vegetation restoration time, whereas Pseudomonadota exhibited the opposite trend, showing a gradual increase. Notably, in addition to bacteria, the top 20 microbial taxa also included Thermoproteota (Archaea), Nitrososphaerota (Archaea), Ascomycota (Fungi), and Basidiomycota (Fungi), although their relative abundances were consistently below 0.50%. Furthermore, a substantial proportion of unclassified microorganisms was identified across samples, with their relative abundance exceeding that of Actinomycetota. On average, unclassified microorganisms accounted for 37.51% of the microbial community, with a maximum value of 46.92%. At the class level, Actinomycetes and Alphaproteobacteria dominated the bacterial community, with their combined average relative abundances exceeding 44.78%. Three archaeal classes, namely Eurotiomycetes, Sordariomycetes, and Dothideomycetes, were identified, with average relative abundances ranging from 0.05 to 0.07%. Only one fungal class, Nitrososphaeria, was identified, with a relatively low average abundance of 0.08%. Unlike the trends observed at the phylum and class levels, the top 20 taxa at the genus and species levels were exclusively bacterial. These taxa were predominantly classified as “unclassified” or “Others,” with the relative abundance of individual taxa not exceeding 10.00%. Among the identified taxa, members of the genus Geodermatophilus were the most abundant, accounting for an average of 8.91%.

**Figure 4:**
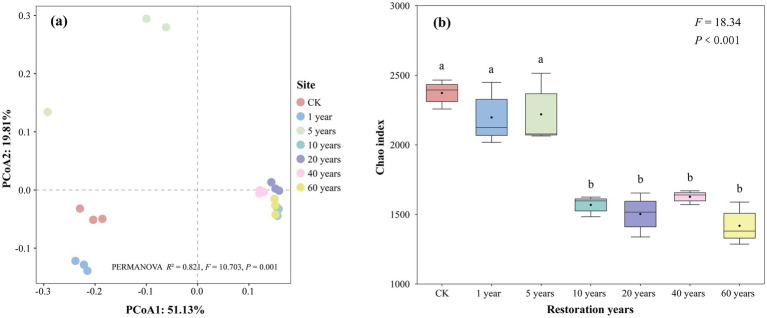
Changes in soil microbial diversity across different stages of vegetation restoration in the study area. **(a)** Principal Coordinates Analysis (PCoA) of soil microbial community composition across different samples; **(b)** variation in the Chao index of soil microbial diversity among different samples.

Diversity analysis revealed that vegetation restoration significantly influenced the composition of the regional soil microbial community, with notable differences across restoration stages (Figure 4). Specifically, principal coordinates analysis (PCoA) based on Bray-Curtis distance divided the microbial communities into four distinct clusters: CK, 1 year, 5 years, and the remaining stages (10, 20, 40, and 60 years). Significant differences were observed among treatments (*R*^2^ = 0.821, *p* = 0.001). Notably, the 5-year restoration samples exhibited greater distances and higher dispersion compared to other stages, whereas the 10–60 year restoration samples were closer together and more tightly clustered. Additionally, vegetation restoration led to a significant decline in microbial diversity in the study area. This decline became more pronounced after 5 years of restoration and stabilized from the 10th year onwards.

### 3.3 Changes in microbial carbon fixation pathways at different restoration stages

**Figure 5:**
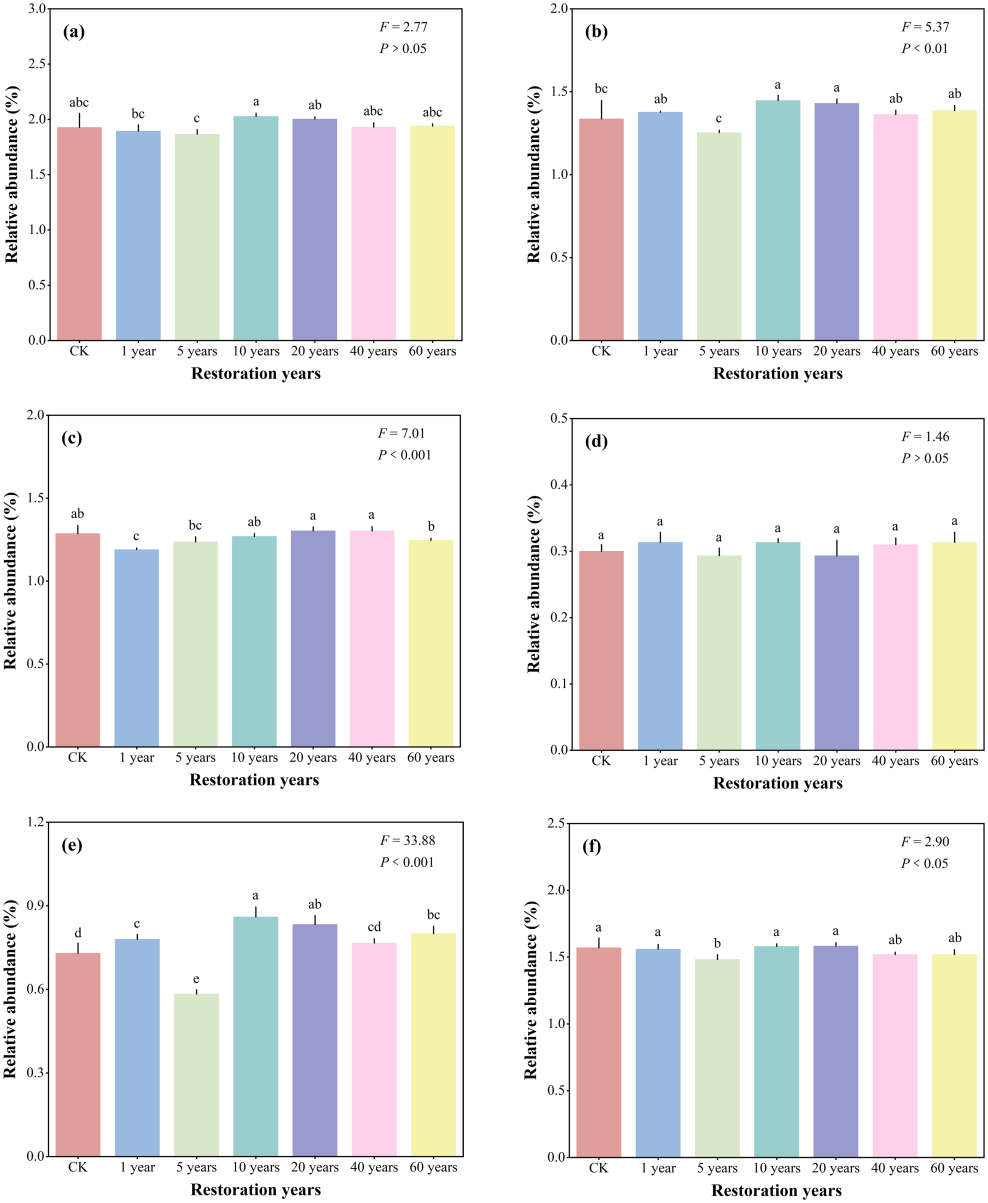
Changes in the relative abundance of six major carbon fixation pathways in soils across different stages of vegetation restoration. **(a)** rTCA cycle, **(b)** 3-HP bi-cycle, **(c)** Calvin cycle, **(d)** WL pathway, **(e)** 3-HP/4-HB cycle, and **(f)** DC/4-HB cycle. Different letters indicate significant differences among vegetation restoration stages (*p <* 0.05), while the same letter indicates no significant difference.

To investigate the changes in soil microbial carbon fixation processes during vegetation restoration, our analysis focused on carbon fixation pathway-related genes. Through metagenomic screening, a total of 90 genes (KOs) involved in carbon fixation pathways were identified. Six major microbial carbon fixation pathways were detected, including the rTCA cycle, 3-HP bi-cycle, Calvin cycle, WL pathway, 3-HP/4-HB cycle, and DC/4-HB cycle (Figure 5).

Among these pathways, the rTCA cycle was identified as the dominant carbon fixation pathway in the region, showing the highest relative abundance, with an average of 1.94% across the sites. The remaining pathways were ranked in descending order of relative abundance as follows: DC/4-HB cycle (1.54%), 3-HP bi-cycle (1.37%), Calvin cycle (1.26%), 3-HP/4-HB cycle (0.76%), and WL pathway (0.31%). Analysis of variance revealed that vegetation restoration significantly altered the relative abundances of the 3-HP bi-cycle, Calvin cycle, 3-HP/4-HB cycle, and DC/4-HB cycle (*p <* 0.05), while its impact on the rTCA cycle and WL pathway was limited. Over the 60-year restoration period, the relative abundances of the rTCA cycle, 3-HP bi-cycle, 3-HP/4-HB cycle, and DC/4-HB cycle exhibited similar trends, characterized by a sharp decrease during the 1–5 year stage, a substantial increase during the 5–10 year stage, and eventual stabilization during the 10–60 year stage. With the exception of the 3-HP/4-HB cycle, the relative abundances of the other three pathways showed minimal variation after 10 years of restoration, with no significant differences among sites (*p* > 0.05). In contrast, the 3-HP/4-HB cycle exhibited more dynamic changes, with a sharp decline followed by a slight recovery during the 20–60 year stage. The Calvin cycle displayed a unique unimodal pattern, gradually increasing during the 1–40 year stage and sharply decreasing during the 40–60 year stage. Although fluctuations in the WL pathway were observed across restoration stages, the differences among sites were not statistically significant. Furthermore, when comparing CK with artificial *Haloxylon* sites, it was found that, except for the 3-HP/4-HB cycle, the relative abundances of the other five pathways differed significantly from CK during the first 10 years of restoration but became similar to CK after 10 years.

**Figure 6:**
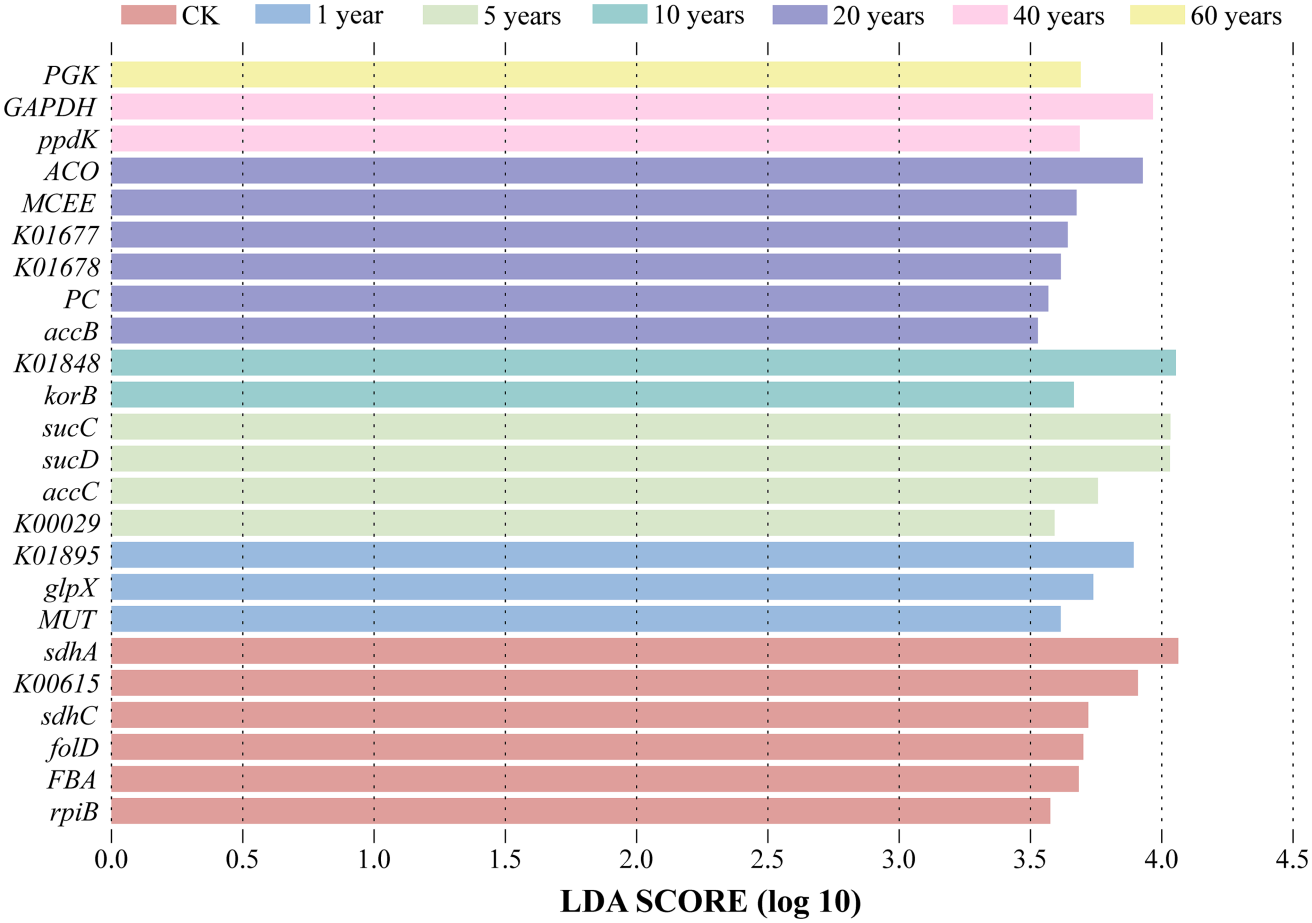
LEfSe analysis of carbon fixation-related genes across different vegetation restoration stages in the study area.

To further elucidate the effects of vegetation restoration on carbon fixation pathways, LEfSe analysis (LDA threshold = 3.5) was performed to identify genes with significant differences in relative abundance among sites (Figure 6). A total of 24 differential genes were identified across the seven sites, with the number of differential genes ranked in descending order as follows: CK (6 genes), 20 years (6 genes), 5 years (4 genes), 1 year (3 genes), 10 years (2 genes), 40 years (2 genes), and 60 years (1 gene). In CK, six genes, including *K00615*, *sdhC*, *folD*, *FBA*, *rpiB*, and *sdhA*, were identified. Among these, genes associated with the Calvin cycle included *K00615*, *FBA*, and *rpiB*. Notably, *sdhA* and *sdhC* participated in both the rTCA cycle and the 3-HP and DC/4-HB cycles, while *folD* was exclusively involved in the WL pathway. In the 1-year restoration site, three genes, *K01895*, *glpX*, and *MUT*, were annotated. These genes were each involved in a single cycle, specifically the WL pathway, Calvin cycle, and 3-HP cycle, respectively. In the 5-year restoration site, four genes, *sucC*, *sucD*, *accC*, and *K00029*, were identified. Among these, *sucC* and *sucD* were jointly involved in both the rTCA cycle and DC/4-HB cycle, while *accC* and *K00029* were associated with the 3-HP cycle and the Calvin-Benson-Bassham (CBB) cycle, respectively. In the 10-year restoration site, only two genes, *K01848* and *korB*, were identified. *K01848* was involved in both the 3-HP cycle and 3-HP/4-HB cycle, whereas *korB* was primarily associated with the rTCA cycle. For the 20-year restoration site, a relatively larger number of differential genes were identified. Genes associated with single cycles included *ACO* (rTCA), *PC* (rTCA), and *accB* (3-HP). In contrast, genes such as *MCEE* (3-HP, 3-HP/4-HB), *K01677* (rTCA, DC/4-HB), and *K01678* (rTCA, DC/4-HB) were involved in multiple cycles. In the 40-year and 60-year restoration sites, fewer differential genes were identified, with most associated with the Calvin cycle. Unlike *PGK* and *GAPDH*, the gene *ppdK* was involved in both the Calvin cycle and the rTCA cycle.

### 3.4 Response of soil microbial communities and functional genes to soil properties

**Figure 7:**
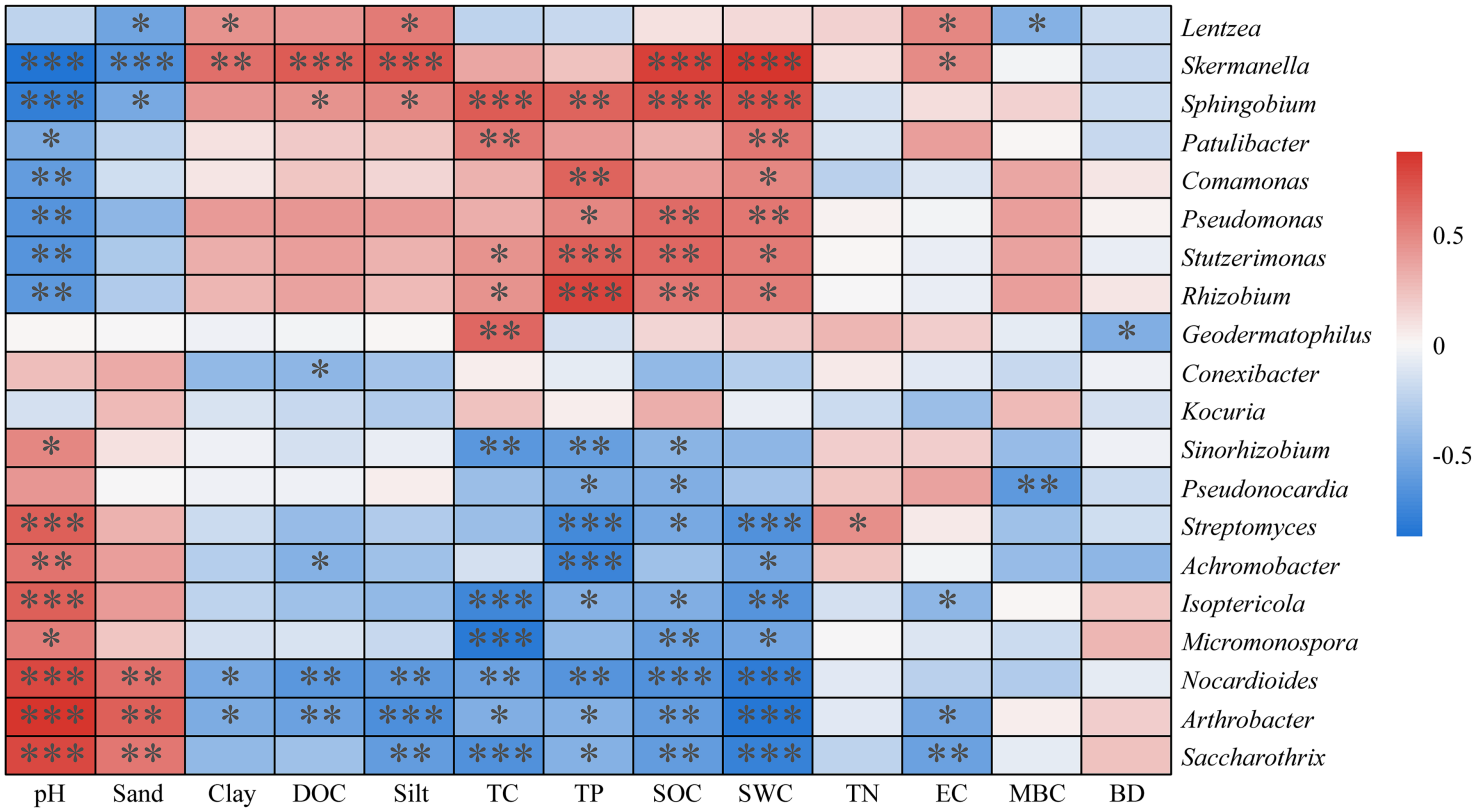
Correlation analysis between soil environmental factors and the top 20 microbial taxa at the genus level. Significance levels are indicated as *p < 0.05, **p < 0.01, and ***p < 0.001.

Correlation analysis revealed varying degrees of association between soil environmental factors and microbial communities (Figure 7). Among the top 20 dominant microorganisms at the genus level, pH, SWC, SOC, and TP were identified as the primary factors influencing relative abundance, whereas TN, BD, and MBC had minimal effects. Specifically, genera such as Kocuria, Conexibacter, and Geodermatophilus were less influenced by soil environmental factors, while Skermanella, Sphingobium, Nocardioides, Arthrobacter, and Saccharothrix were strongly affected. Notably, pH showed a highly significant positive correlation (*p <* 0.01) with genera such as Arthrobacter, Saccharothrix, Nocardioides, Streptomyces, and Isoptericola, and a highly significant negative correlation (*p <* 0.01) with Skermanella and Sphingobium. Conversely, SWC, SOC, and TP exerted effects opposite to those of pH on the aforementioned genera.

**Figure 8:**
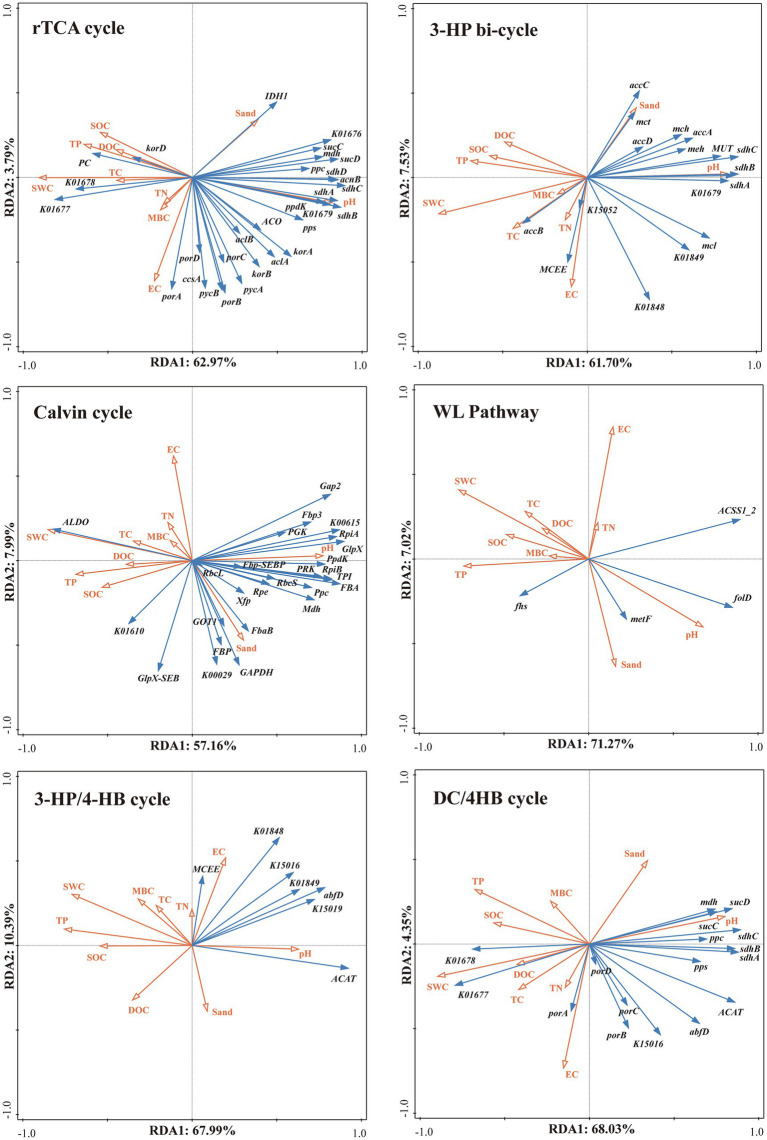
Redundancy analysis (RDA) of soil properties and carbon fixation-related genes across six pathways in the study area.

Redundancy analysis (RDA) was used to explore the effects of soil physicochemical properties on the abundances of key genes associated with six carbon fixation pathways. The results indicated that SWC was the most important environmental factor influencing the abundances of carbon fixation pathway genes, significantly affecting all six pathways (*p <* 0.01). TP exhibited varying degrees of influence on different carbon fixation pathways: it had a highly significant effect (*p <* 0.01) on the WL pathway, 3-HP/4-HB cycle, and DC/4-HB cycle, and a significant effect (*p <* 0.05) on the rTCA cycle, 3-HP bi-cycle, and Calvin cycle. Additionally, EC significantly influenced the 3-HP/4-HB cycle (*p <* 0.05). In contrast, factors such as TN, DOC, Sand, and MBC had no significant effects (Table 4; Figure 8).

**Table 4:** Contributions of soil properties to the changes in soil carbon fixation-related genes across different vegetation restoration stages.

Soil properties	rTCA cycle		3-HP bi-cycle		Calvin cycle		WL pathway		3-HP/4-HB cycle		DC/4-HB cycle	
	Contribution %	*P*	Contribution %	*P*	Contribution %	*P*	Contribution %	*P*	Contribution %	*P*	Contribution %	*P*
SWC	72.8	﹤0.01	65.2	﹤0.01	58.4	﹤0.01	54.1	﹤0.01	19.3	﹤0.01	72.8	﹤0.01
TP	10.4	﹤0.05	14.0	﹤0.05	14.2	﹤0.05	7.4	﹤0.01	48.0	﹤0.01	13.7	﹤0.01
EC	1.9	n.s.	4.9	n.s.	5.0	n.s.	26.6	n.s.	11.7	﹤0.05	2.2	n.s.
pH	2.5	n.s.	3.7	n.s.	2.6	n.s.	1.5	n.s.	4.9	n.s.	2.3	n.s.
TC	2.4	n.s.	3.0	n.s.	4.0	n.s.	1.4	n.s.	5.7	n.s.	2.0	n.s.
Sand	2.3	n.s.	1.4	n.s.	3.0	n.s.	1.2	n.s.	1.4	n.s.	1.1	n.s.
MBC	1.5	n.s.	2.3	n.s.	3.6	n.s.	1.3	n.s.	2.2	n.s.	0.8	n.s.
SOC	3.8	n.s.	3.5	n.s.	3.8	n.s.	1.8	n.s.	1.9	n.s.	2.8	n.s.
DOC	1.3	n.s.	1.1	n.s.	2.9	n.s.	3.5	n.s.	2.6	n.s.	1.2	n.s.
TN	1.0	n.s.	0.9	n.s.	2.3	n.s.	1.3	n.s.	2.4	n.s.	1.1	n.s.

If the significance level (*p*-value) of the soil property contribution is <0.05, the soil property is considered a major factor influencing the variation in the abundance of regional carbon sequestration-related genes. Conversely, when the significance level (*p*-value) of the soil property contribution is greater than or equal to 0.05 (n.s.), it is considered to have no significant effect.

In the rTCA cycle, SWC and TP were positively correlated with genes such as *PC*, *K01677*, *K01678*, and *korD*, but negatively correlated with genes including *sucD*, *sdhA*, and *mdh*. Similar to the rTCA cycle, SWC and TP exhibited negative correlations with most genes in the 3-HP bi-cycle, except for a few, such as *accB* and *K15052*, which showed positive correlations. The strongest negative correlations were observed for genes such as *sdhB*, *mcl*, and *meh*. In the Calvin cycle (CBB pathway), only *ALDO* and *K01610* were positively influenced by SWC and TP, while most other genes showed negative correlations. The WL pathway, which had fewer annotated genes, exhibited a mixture of effects: SWC had a negative impact on genes such as *metF*, *folD*, and *ACSS1_2*, but a positive effect on *fhs*, with TP showing similar trends. Beyond SWC and TP, EC also significantly influenced the abundances of genes in the 3-HP/4-HB cycle, showing positive correlations with several key genes, such as *abfD* and *MCEE*. Although numerous genes were annotated in the DC/4-HB cycle, most showed negative correlations with SWC and TP. Among the 17 genes identified, only *K01677* and *K01678* exhibited significant positive correlations with SWC and TP.

## 4 Discussion

Our results show that, compared with mobile sandy land, the contents of soil organic carbon (SOC), total nitrogen (TN), and total phosphorus (TP) in artificial *Haloxylon* sites with clay sand barriers were notably increased. This finding is consistent with previous studies, further confirming that vegetation restoration significantly enhances the carbon and nitrogen pools in desert soils (Ma et al., 2021). After the establishment of *Haloxylon* as the dominant species in the community, its tall plants not only effectively reduce wind erosion but also intercept and accumulate dust particles from aeolian sediment and forest litter, playing a positive role in the enrichment of soil carbon and nitrogen reserves (Su et al., 2010). In fact, various nutrients, particularly SOC and TN, are rarely derived from the soil parent material but instead originate from particle deposition and the metabolic processes of the vegetation itself (Fan et al., 2016). As vegetation restoration progresses, the TP content also increases, although its growth rate remains relatively limited. Previous studies have demonstrated that the effects of *Haloxylon* establishment on soil TP content are relatively minor, with significant increases in TP typically observed only after 40 years of restoration (Su and Liu, 2020). Furthermore, the arid, low-precipitation environment of the study area, combined with strong wind erosion, hinders the rapid decomposition of litter, exacerbating the loss of soil carbon and nitrogen pools. Additionally, the decline of *Haloxylon* observed in some mature forests reduces the amount of nutrients returned to the soil, collectively leading to the relatively low soil carbon storage observed in the region (Ma et al., 2017). Our data also indicate that vegetation restoration influences soil clay content (Clay), soil water content (SWC), electrical conductivity (EC), and pH. This may be attributed to the increased productivity of shrubs and the stabilization of shrub clusters, which reduce the damage caused by wind erosion to surface soil, gradually decreasing sand particle content while increasing clay and silt particle content (Xiao et al., 2003). The production of litter and the addition of organic matter further improve soil texture. Although shrubs consume substantial amounts of soil water during their growth, the expansion and maturation of *Haloxylon* stems, leaves, and root systems not only reduce water evaporation and transpiration but also facilitate the upward transport of deep soil water or groundwater to the surface soil, partially alleviating water stress on plants (Kidron and Gutschick, 2013). The increase in soil EC is primarily due to the accumulation of salts, which lowers the osmotic potential and water potential of plant tissues, thereby enhancing the plants’ ability to absorb water and improve drought resistance. Additionally, the establishment of artificial *Haloxylon* forests effectively mitigates soil alkalinity, a conclusion supported by the observed changes in soil pH in this study (Xi et al., 2015; Wang et al., 2023).

In general, vegetation restoration promotes the accumulation of soil organic matter and nutrients, which in turn enhances microbial diversity and functionality (Araujo Pereira et al., 2021). However, in arid regions, relatively scarce resources strongly constrain soil microbial metabolism and composition, potentially weakening their support for soil functionality (Gunnigle et al., 2017). In this study, as vegetation restoration progressed, the relative abundance of soil bacteria remained relatively stable, fungal abundance gradually declined, and archaeal abundance exhibited a fluctuating upward trend. These patterns are primarily related to microbial adaptability. Bacteria, being the dominant group in the study area, possess high functional redundancy, which allows them to maintain stability under changing soil conditions. Additionally, their rapid reproduction capacity enables bacteria to respond more swiftly to environmental fluctuations, quickly rebalancing community structure (Xue et al., 2023). In arid regions, fungi often serve as the primary functional group responsible for decomposing complex organic matter in nutrient-poor soils. However, as vegetation restoration progresses, the accumulation of easily decomposable organic matter in soil reduces the relative importance of fungi (Hartman et al., 2017). Meanwhile, many archaea are better adapted to neutral or slightly acidic environments, and the gradual shift in soil pH from alkaline to neutral in the later stages of restoration supports archaeal proliferation over the long term (Clouet-d’ Orval et al., 2018).

We observed that soil microbial diversity in the study area underwent a pronounced decline during the 5–10 year restoration phase, gradually stabilizing thereafter, yet remaining significantly lower than that observed in the control sandy soils. This finding contrasts with the commonly accepted conclusion that soil microbial diversity increases with vegetation restoration (Hu et al., 2024). One possible explanation is that the absence of vegetation cover in mobile sandy land creates a nutrient-poor environment with minimal resource competition, allowing more microbial species to coexist through niche differentiation (Hartman et al., 2017). In the early stages of vegetation restoration (0–10 years), the expansion of plant roots and the accumulation of litter significantly alter soil nutrient levels and microclimatic conditions. This enrichment of resources may intensify microbial competition, leading to the displacement of weaker species by dominant populations, thereby causing a marked reorganization of the microbial community (Kuzyakov and Blagodatskaya, 2015). After 10 years of restoration, as the vegetation community stabilizes, the rate of organic matter input and environmental changes slows, and the microbial community’s function and composition also reach equilibrium. Another possible explanation relates to vegetation diversity. According to the “cumulative complementarity effect” in macroecology, as plant community succession progresses, the impact of vegetation diversity on microbial biomass and functionality shifts from an initial negative effect to a positive effect in later stages (Zhou et al., 2024). Previous studies on regional vegetation communities showed that vegetation diversity indices (Shannon-Wiener, Simpson, and Pielou) significantly increased during the 0–5 year stage, peaked at the 5-year mark, and then sharply declined, stabilizing after 10 years (Song et al., 2024). Influenced by the nutrient limitations of arid soils, strong nutrient competition between plants and microbes may occur during the early stages of vegetation restoration. Plants, as the victorious competitors, often suppress microbial biomass and functionality. This explains why microbial biomass carbon (MBC) decreased during the 0–1 year stage, increased sharply between 5–10 years, and stabilized after 20 years. In this study, MBC reached its peak at the 10-year restoration site; however, the relatively large standard deviation may be mainly attributed to the pronounced spatial heterogeneity of soil microbial communities during the 5- to 10-year restoration stage. Furthermore, previous research has demonstrated that community stability and microbial diversity may exhibit a negative correlation. Moderate reductions in microbial diversity can enhance interactions between network-classified OTUs and increase modularity, thereby improving the metabolic efficiency and stability of the microbiome (Yang et al., 2023).

We investigated the phylogenetic affiliations of microbial communities across different vegetation restoration stages and found that the soil in the study area was dominated by Actinomycetota (Actinobacteria) and Pseudomonadota (Proteobacteria), which are the predominant microbial groups in the region. This finding is consistent with studies conducted in the Horqin Sandy Land and the Mu Us Sandy Land (Sun et al., 2020). Actinomycetota and Pseudomonadota exhibit high adaptability to arid environments and play crucial roles in soil carbon fixation (Fierer et al., 2007). However, their trends over restoration years varied. As an oligotrophic microorganism, Actinomycetota demonstrates significant resilience in harsh environments, contributing to the decomposition of complex organic compounds and organic matter. Its abundance, however, decreased significantly with the extension of artificial vegetation restoration (Sibanda et al., 2019). In contrast, Pseudomonadota, categorized as copiotrophic bacteria, thrives in nutrient-rich soil environments (Fierer et al., 2012). During the early stages of vegetation restoration, the relatively low soil nutrient content limited the proliferation of Pseudomonadota. As soil nutrient levels increased over time, more favorable conditions were created for their growth and expansion. The contrasting trends in Actinomycetota and Pseudomonadota abundance highlight a broader shift in the soil microbial community composition, transitioning from oligotrophic to copiotrophic groups as vegetation restoration progresses. Additionally, the relatively high abundance of unclassified microorganisms in the regional soil samples indicates the existence of a substantial proportion of microbial resources that remain underexplored, potentially including microbial taxa specific to desert environments. The high proportion of unclassified microorganisms also reflects the complexity of the soil microbial community in the study area. Research confirms that arid regions harbor distinct microbial communities that play a major role in elemental cycling, warranting further investigation (Yang et al., 2024).

Through gene function annotation, we identified that all six major carbon fixation pathways were annotated in the soil microbiomes of the study area. This result is consistent with findings from similar desert ecosystems (Zhang et al., 2024). In this study, the rTCA cycle exhibited the highest relative abundance, making it the most dominant microbial carbon fixation pathway in the region, in contrast to the Calvin cycle, which is commonly reported as dominant in other studies (Paul et al., 2000). The primary carbon fixation pathways in desert soils often differ from those in other habitats, as carbon-fixing microorganisms in arid regions utilize different pathways depending on environmental conditions (Yang et al., 2024). In deserts, the limited energy supply in soils plays a crucial role, as the energy demands of various carbon fixation pathways differ. As a result, energy utilization efficiency becomes particularly important. Only pathways with low energy requirements and high energy efficiency—such as the rTCA cycle and WL pathway—can maintain relatively high abundances in CO₂ fixation under such conditions (Bar-Even et al., 2012). In contrast, the Calvin cycle is likely suppressed by the harsh conditions of sandy soils, including intense solar radiation and extremely low moisture content (Costello et al., 2009). Furthermore, the functional inertia engendered within microbial communities under prolonged drought stress precludes the energetically expensive Calvin cycle from supplanting the established dominant populations over short temporal scales (Broderick et al., 2025). It is worth noting that while low-energy pathways ensure higher productivity, they often result in lower growth rates (Wortel et al., 2018). Additionally, some key enzymes involved in the WL pathway exhibit high sensitivity to oxygen (Liu, 2019). The sandy soil in the study area may fail to meet the strict anaerobic conditions required for these enzymes, which likely explains why the WL pathway displayed the lowest relative abundance in this study. We also observed that the relative abundances of the 3-HP bi-cycle, 3-HP/4-HB cycle, and DC/4-HB cycle were significantly lower in soils during the 5-year restoration stage compared to other restoration stages, followed by a sharp increase afterward. This trend can be attributed to the gradual increase in soil nutrients driven by vegetation restoration, as a more abundant energy supply benefits microorganisms utilizing carbon fixation pathways with higher energy demands, such as the 3-HP bi-cycle and 3-HP/4-HB cycle. Additionally, some mixotrophic microorganisms that rely on the 3-HP bi-cycle improve their carbon fixation capacity as nutrient availability increases (Hügler and Sievert, 2011). Notably, among the six carbon fixation pathways, the 3-HP/4-HB cycle exhibited the largest fluctuations in relative abundance. Unlike multipurpose pathways such as the Calvin cycle, the 3-HP/4-HB cycle has higher energy requirements and responds more sensitively to environmental changes. During the early stages of vegetation restoration, unstable soil conditions favored microorganisms utilizing other carbon fixation pathways (Calvin cycle), which adapted more quickly to environmental changes and established dominance. However, between 5–10 years, as the *Haloxylon* community stabilized and soil organic matter and nutrient levels significantly improved, the microbial community structure began to rebalance. At this stage, microorganisms dependent on the 3-HP/4-HB cycle gradually adapted to the improved soil environment and restored their ecological functions. In the later stages of vegetation restoration (> 10 years), as soil conditions became increasingly stable, microbial community structure and functional diversity reached a new equilibrium. During this period, the microbial communities relying on the 3-HP/4-HB cycle maintained stability within their ecological niche, and the fluctuations in their relative abundance became less pronounced.

The functional genomics of surface soil microorganisms are significantly influenced by environmental changes (Bahram et al., 2018). In the study area, the gene abundances of different carbon fixation pathways also varied across vegetation restoration stages. Specifically, in the 1-year restoration site, genes such as *K01895* (involved in the WL pathway), *glpX* (Calvin cycle), and *MUT* (3-HP bi-cycle) were identified as exclusively participating in individual carbon fixation pathways, with highly specialized functions. This indicates that carbon-fixing microorganisms in the early stages of restoration had limited adaptive capacities and exhibited pronounced functional differentiation. In the 5-year restoration site, the differential genes detected (*sucC* and *sucD*) were involved in multiple carbon fixation pathways, such as the rTCA cycle and DC/4-HB cycle, suggesting that microbial carbon fixation functions had begun to diversify but were still primarily concentrated in core pathways adapted to harsh environments. In the 20-year restoration site, multi-pathway genes (*K01677* and *K01678*) associated with the rTCA cycle and DC/4-HB cycle demonstrated greater redundancy and adaptability, indicating that these pathways contributed significantly to soil carbon fixation during the mid-stage of restoration. However, by the 60-year restoration stage, only one differential gene, *ppdK*, was detected, suggesting that soil carbon fixation functions had become increasingly stable in the later stages of restoration. Overall, over the 60 years of vegetation restoration, although the number of differential carbon fixation genes decreased, their functional redundancy increased, as evidenced by more pronounced synergistic interactions among different carbon fixation pathways. This trend is likely associated with microbial community differentiation and selection over the long-term vegetation restoration process.

The structure of soil bacterial communities is significantly influenced by surface vegetation and soil environmental factors (Dong et al., 2023). Vegetation alters soil physicochemical properties through the decomposition and differentiation of litter and root systems, while soil bacterial communities are closely related to these physicochemical factors (Falkowski et al., 2008). In the study area, the main factors affecting microbial community structure were pH, soil water content (SWC), soil organic carbon (SOC), and total phosphorus (TP), which aligns with the common understanding derived from research in multiple desertification control areas across China (Deng et al., 2020). Specifically, in this study, pH showed a highly significant positive correlation (*p <* 0.01) with genera such as Arthrobacter, Saccharothrix, Nocardioides, Streptomyces, and Isoptericola, indicating that these genera are well-adapted to the region’s alkaline soil environment. In contrast, pH exhibited a highly significant negative correlation (*p <* 0.01) with Skermanella and Sphingobium, suggesting that changes in soil pH caused by vegetation restoration could inhibit the growth of these genera, thereby altering the composition of the microbial community. Unlike pH, SWC, SOC, and TP negatively affected the relative abundances of genera such as Arthrobacter and Saccharothrix, while having a positive effect on Skermanella and Sphingobium. This bidirectional influence indicates that improvements in soil properties, such as increased moisture content and nutrient levels, do not universally benefit all microorganisms. On the contrary, they may suppress certain microbes due to increased competition, particularly in arid regions. Additionally, some genera, such as Kocuria, Conexibacter, and Geodermatophilus, exhibited low sensitivity to soil changes, suggesting that these microorganisms possess strong adaptability to environmental fluctuations. These resilient microbes play a crucial role in maintaining the functional integrity of soil microbial communities during vegetation restoration.

Previous studies have shown that the relative abundance of carbon cycle-related genes typically increases with rising SOC levels or as soil pH approaches neutrality. However, this pattern does not align with the findings of this study (Dong et al., 2023; Guo et al., 2022). In the study area, SOC, total nitrogen (TN), and total phosphorus (TP) levels increased progressively with restoration age, and soil pH gradually approached neutrality. Yet, the abundance of carbon fixation pathway-related genes stabilized after 10 years and did not continue to increase. Furthermore, redundancy analysis revealed that SOC, SWC, and TP were negatively correlated with the relative abundance of most carbon fixation genes. One possible explanation is that SOC accumulation does not rely solely on microbial autotrophic carbon fixation pathways but is instead driven by multiple ecological processes (Wang et al., 2019). During the early stages of vegetation restoration, the soil microbial community was dominated by autotrophic microbes with strong carbon fixation capabilities (Nitrososphaeria). As restoration progressed, the input of plant-derived organic matter (litter and root exudates) increased, leading to the gradual dominance of heterotrophic microbes capable of decomposing complex organic matter (Pseudomonadota). The observed decline in Nitrososphaeria and increase in Pseudomonadota in this study support this hypothesis. In the later stages of vegetation restoration, SOC accumulation likely depends more on plant carbon input and the decomposition activity of heterotrophic microbes rather than microbial autotrophic carbon fixation. We define this phenomenon as a “functional substitution” mechanism, whereby the mechanism of soil carbon pool expansion gradually shifts from dependence on microbial autotrophic carbon fixation to heterotrophic metabolism driven by plant carbon inputs during vegetation restoration. Additionally, the negative effects of SWC and TP on carbon fixation genes suggest that increases in SOC and TP provide abundant resources for heterotrophic microbes, which, while promoting their growth, may suppress the activity of autotrophic microbes, thereby reducing the relative abundance of carbon fixation genes.

Another possible explanation is that microbial communities are more constrained by soil moisture and carbon availability in the early stages of vegetation succession, whereas phosphorus becomes the primary limiting factor in the later stages (Philippot et al., 2024). The relatively low soil moisture and SOC levels observed during the early stages of succession in this study support this hypothesis. In the early restoration phase, vegetation recovery consumes significant amounts of water, and the relatively arid soil conditions drive the evolution of microbial strategies to tolerate drought and salinity-alkalinity. While long-term vegetation restoration re-establishes a new dynamic balance in soil moisture, these drought-tolerance strategies may not shift rapidly and may even hinder the carbon fixation potential of microbes. Most of the phosphorus required by microbes is derived from the decomposition of soil organic matter (Tarafdar and Claassen, 1988). In the later stages of vegetation restoration, the underground root system’s phosphorus uptake reduces the availability of phosphorus in soil, creating competition between plants and microbes for this nutrient (Inselsbacher et al., 2010). The gradual decrease in soil pH improves the availability of complex phosphorus compounds, making phosphorus more accessible to microbes and plants in alkaline soils. Additionally, studies have shown that under phosphorus limitation, microbial communities may consume more carbon and nitrogen to produce and secrete phosphorus-metabolizing enzymes (Marklein and Houlton, 2012). From this perspective, phosphorus limitation may enhance the carbon metabolic efficiency of microbes in the later stages of vegetation succession.

Our study has several limitations. A large proportion of microbial taxa remained unclassified, and these unknown species may harbor unique carbon fixation functions. Moreover, metagenomic analysis reveals gene presence at the DNA level rather than actual functional expression. Future studies should combine meta-transcriptomics and metabolomics to validate the functional roles of key microbial groups and to distinguish active carbon fixers from dormant populations, thereby providing a more accurate understanding of microbial carbon sequestration mechanisms in this arid ecosystem. Despite these limitations, our findings offer broader insights into the coupled interactions among soil properties, microbial community dynamics, and carbon fixation processes. We demonstrate that vegetation restoration simultaneously modifies soil physicochemical conditions, restructures microbial assemblages. The persistence of the low-energy rTCA cycle under water-limited conditions, coupled with the “functional substitution” mechanism, highlights the intertwined abiotic-biotic controls on soil carbon dynamics in arid systems. These results provide a theoretical basis for predicting microbial carbon responses to ecological restoration in global drylands.

## 5 Conclusion

This study provides the first metagenomic investigation of how clay sand barriers and artificial Haloxylon restoration influence soil microbial carbon fixation pathways in arid desert regions of northwest China. Our results indicate that *Haloxylon* restoration with clay sand barriers improves soil nutrient levels and organic matter accumulation, with these positive effects strengthening over the 60-year restoration time sequence. Vegetation restoration significantly altered soil microbial community composition and drove changes in carbon fixation-related genes and pathways, with the most pronounced shifts occurring within the first 10 years, followed by relative stabilization. The rTCA cycle was identified as the dominant carbon fixation pathway in this region. Soil water content (SWC) and total phosphorus (TP) were the key factors driving variations in both microbial community composition and carbon fixation gene abundance, while pH, soil organic carbon (SOC), and electrical conductivity (EC) also contributed to specific aspects of these changes. These findings highlight the crucial role of clay sand barrier-*Haloxylon* restoration in microbially mediated soil carbon sequestration, offering practical guidance for ecological engineering and carbon management in arid desert ecosystems.

## Data Availability

The original contributions presented in the study are publicly available. The raw metagenomic sequencing data have been deposited in the Genome Sequence Archive (GSA) of the China National Center for Bioinformation (CNCB) under accession number CRA046182 (https://ngdc.cncb.ac.cn/gsa).
